# The oncogenic receptor ErbB2 modulates gemcitabine and irinotecan/SN-38 chemoresistance of human pancreatic cancer cells *via* hCNT1 transporter and multidrug-resistance associated protein MRP-2

**DOI:** 10.18632/oncotarget.3414

**Published:** 2015-03-26

**Authors:** Nicolas Skrypek, Romain Vasseur, Audrey Vincent, Bélinda Duchêne, Isabelle Van Seuningen, Nicolas Jonckheere

**Affiliations:** ^1^ Inserm, UMR-S1172, Jean Pierre Aubert Research Center, Team #5 “Mucins, epithelial differentiation and carcinogenesis”, Lille cedex 59045, France; ^2^ Université Lille Nord de France, Lille cedex 59000, France; ^3^ Centre Hospitalier Régional et Universitaire de Lille, Lille cedex 59037, France

**Keywords:** ErbB2, pancreatic cancer, gemcitabine, FOLFIRINOX, chemoresistance

## Abstract

Pancreatic adenocarcinoma (PDAC) is one of the most deadly cancers because of a lack of early diagnotic markers and efficient therapeutics. The fluorinated analog of deoxycytidine, gemcitabine and emerging FOLFIRINOX protocol (5-fluorouracil (5-FU), irinotecan/SN-38, oxaliplatin and leucovorin) are the main chemotherapies to treat PDAC. The ErbB2/HER2 oncogenic receptor is commonly overexpressed in PDAC. In this context, we aimed to decipher the ErbB2-mediated mechanisms of chemoresistance to the two main chemotherapy protocols used to treat PDAC.

ErbB2 knocking down (KD) in CAPAN-1 and CAPAN-2 cells led to an increased sensitivity to gemcitabine and an increased resistance to irinotecan/SN-38 both *in vitro* and *in vivo* (subcuteanous xenografts) This was correlated to an increase of hCNT1 and hCNT3 transporters and ABCG2, MRP1 and MRP2 ATP-binding cassette transporters expression and resistance to cell death. We also show that MRP2 is repressed following activation of JNK, Erk1/2 and NF-κB pathways by ErbB2. Finally, in datasets of human PDAC samples, ErbB2 and MRP2 expression was conversely correlated. Altogether, we propose that ErbB2 mediates several intracellular mechanisms linked to PDAC cell chemoresistance that may represent potential targets in order to ameliorate chemotherapy response and allow stratification of patients eligible for either gemcitabine or FOLFIRINOX treatment.

## INTRODUCTION

Pancreatic adenocarcinoma (PDAC) is one of the most deadly cancers in western countries with an extremely poor prognosis (survival rate of 6 months) [1, 2]. Pancreatic cancers are projected to become the second leading cause of cancer-related death by 2030 [3]. This dramatic outcome is related to a lack of efficient therapeutic tools and early diagnostic markers. At the time of diagnosis, more than 80% of patients have metastasis or locally advanced cancer. Only about 10 to 15% of patients are considered eligible for surgical resection. Gemcitabine, a fluorinated analog of deoxycytidine, is the main chemotherapeutic drug used in firstline in advanced pancreatic cancer (PC). Despite the improvement of patient's quality of life, the gain in survival remains short (6 additional months). This is mainly due to the high resistance of PC cells to the drug [4]. In 2011, the FOLFIRINOX regimen (5-fluorouracil (5-FU), leucovorin, irinotecan, and oxaliplatin) emerged as a new option in patients with metastatic PC and a good performance status [5]. FOLFIRINOX was associated with a survival advantage but had increased toxicity. SN-38 which is the active metabolite of irinotecan binds to the topoisomerase I thereby (i) inducing DNA cleavage during S phase of cell cycle and (ii) driving apoptosis [6]. Among SN-38-mediated chemoresistance mechanisms is the inactivation of SN-38 by UDP-glucuronosyl transferase in the liver [7]. Another mechanism related to chemoresistance is the increase of multidrug resistance protein MDR1 (P-glycoprotein/ABCB1), MDR-related proteins MRP1/2 (ABCC1/2), or ABCG2 (BCRP) ATP-binding cassette transporter expression [7]. These transporters play important roles in normal physiology by exporting toxic xenobiotics as well as many drugs used in clinics and thereby reducing their efficiency [8].

Deciphering the mechanisms responsible for PDAC cell resistance to chemotherapy is thus mandatory if one wants to improve efficacy of the drugs and propose more efficient therapies.

The ErbB2/HER2 type I transmembrane growth factor receptor belongs to the epidermal growth factor receptor (EGFR/ErbB1) family also comprising ErbB3 and ErbB4. The ErbB2 protein consists in an extracellular domain, a transmembrane domain and an intracellular tyrosine kinase domain. ErbB2 has no known ligand and is described as a coreceptor which hetero-dimerizes with the other ErbB receptors [9]. ErbB2 is commonly overexpressed and frequently amplified in cancers, including PDAC [10]. Notably, ErbB2 overexpression was shown to be an independent factor for a worse prognosis in PDAC [11]. The MUC4 membrane-bound mucin, that is a membrane partner of ErbB2 [12], was recently shown to regulate the hCNT1 transporter expression *via* the NF-κB pathway leading to decreased PC cell sensitivity to gemcitabine [13]. Previous studies suggested the relationship of the ErbB2 receptor and chemotherapeutic sensitivity to lapatinib eventually combined with SN-38 in other epithelial cancers [14, 15]. However, the involvement of ErbB2 in chemo-resistance in PDAC remains to be elucidated.

In this work, we demonstrate for the first time that ErbB2 silencing leads to an increased sensitivity of PC cells to gemcitabine *via* hCNT1/3 transporters. Moreover, ErbB2 silencing induces PC cell resistance to SN-38 treatment *via* an upregulation of MRP2 multidrug resistance protein. Finally, we show that ErbB2 and MRP2 expression is conversely correlated in human PDAC samples.

## RESULTS

### Loss of ErbB2 induces PDAC cell sensitivity to gemcitabine and resistance to SN-38

**Figure 1:**
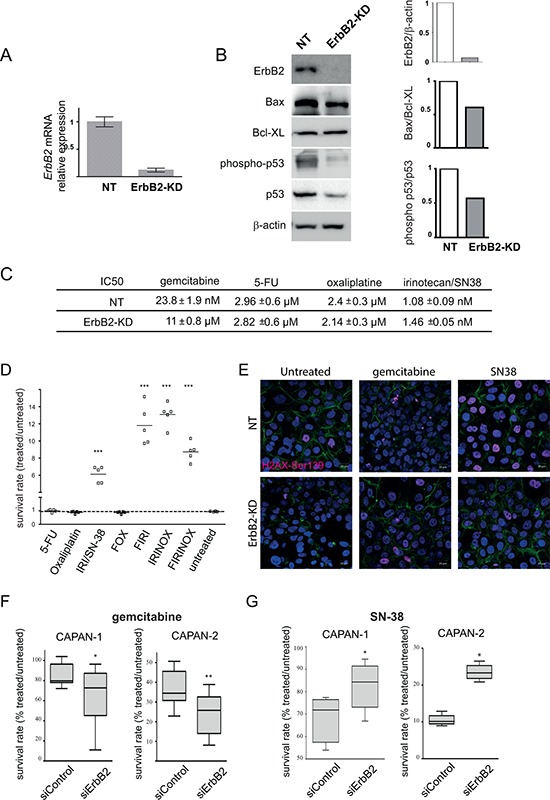
ErbB2 deficient cells are more sensitive to gemcitabine and resistant to SN-38 treatment **A.** mRNA expression of *ErbB2* was analyzed in non targeting (NT) and ErbB2-KD cells by qRT–PCR. The histogram represents the ratio of their expression in ErbB2-KD compared with NT cells. Three independent experiments were performed. **B.** ErbB2, Bax, Bcl_XL_, phospho-p53, p53 and β-actin expression was analysed by western blotting. Bands were quantified by densitometry and ratio of intensities are shown in the histogram. **C.** IC_50_ rates were measured after 72 h of gemcitabine, 5-FU, oxaliplatin or SN-38 irinotecan treatment in ErbB2-KD and control NT cells. **D.** Survival rates in ErbB2-KD or their NT control cells were measured following treatment with different drug combinations using the MTT assay. Three independent experiments were performed. The sensitivity of NT control cells (NT) is arbitrarily reported to 1. FOX = 5-FU (1 μM) + oxaliplatin (1 μM), FIRI = 5-FU + SN-38 (2 nM), IRINOX = SN-38 + oxaliplatin, FIRINOX = 5-FU + SN-38 + oxaliplatin. **E.** Phospho-Ser139 H2A.X foci were visualized by immunofluorescence and confocal microscopy in NT and ErbB2-KD CAPAN-2 cells. Cells were treated with gemcitabine (100 nM) or SN-38 (20 nM) for 24 h. F-actin was stained with Alexa-488 conjugated Phalloidin. Nuclei were stained using DAPI. Three independent experiments were performed. Scale bar = 20 μm. **F–G.** Survival rates in CAPAN-1 and CAPAN-2 cells were measured following transient siRNA-ErbB2 and treatment with gemcitabine (F) or SN-38 (G) using the MTT assay.

We previously generated CAPAN-2 stable cell clones in which ErbB2 was knocked down (ErbB2-KD) by a shRNA approach [12]. ErbB2 knocking down was first confirmed by qRT-PCR (Figure 1A) and western blotting (Figure 1B). The loss of ErbB2 was correlated with a decreased expression of the proapoptotic marker Bax and a mild increased expression of the antiapoptotic marker Bcl_XL_, leading to a decrease of the Bax/Bcl_XL_ ratio, suggesting a lower susceptibility to apoptosis. Additionally, the activation of the cell cycle and apoptosis mediator p53 (phosphorylated p53/constitutive p53 ratio) was decreased in ErbB2-KD cells (Figure 1B).

Measurement of ErbB2-KD sensitivity to gemcitabine was studied in CAPAN-2 cells. IC_50_ showed that the loss of ErbB2 leads to an increased sensitivity of CAPAN-2 cells to gemcitabine (ErbB2-KD = 11 ± 0.8 nM vs NT = 23.8 ± 1.9 nM) (Figure 1C).

The sensitivity to the drugs of the FOLFIRINOX protocol showed no difference for 5-FU (ErbB2-KD = 2.82 ± 0.6 μM vs NT = 2.96 ± 0.6 μM) or oxaliplatin (ErbB2-KD = 2.14 ± 0.3 μM vs NT = 2.4 ± 0.3 μM) and a mild increase of sensitivity to SN-38, the irinotecan active metabolite, (NT 1.08 ± 0.09 nM vs ErbB2-KD 1.46 ± 0.05 nM). Using different combinations of 5-FU, oxaliplatin and SN-38, we show that the lack of ErbB2 potentiates survival to SN-38 alone (6-fold, *p* < 0.001) or combined with 5-FU (FIRI), oxaliplatin (IRINOX) or both molecules (FIRINOX) (Figure 1D). H2A.X phosphorylated at Ser139 is required for DNA repair following double-stranded DNA breaks [16]. SN-38 treatment induced an increase of phospho-Ser139 H2A.X foci in the nuclei of both NT and ErbB2-KD cells and an increase of positive cells (Figure 1E and supplemental Figure S1). This increase was higher in NT cells compared to ErbB2-KD cells following SN-38 treatment suggesting less DNA damage in ErbB2-KD cells. We also observed a mild increase of phospho-Ser139 H2A.X in ErbB2-KD cells following gemcitabine treatment.

By transient inhibition of ErbB2 using a siRNA approach, we observed a decrease of cell survival following gemcitabine treatment of PC cells compared to control (Figure 1F). Moreover, we showed a significant increase of survival rate of both CAPAN-1 and CAPAN-2 cells following SN-38 treatment (Figure 1G). We also confirmed the ErbB2-mediated alteration of chemosensitivity in Panc1 and BxPC-3 cells (supplemental Figure S2). Altogether these results indicate that ErbB2 may be involved in mediating PDAC cell sensitivity to gemcitabine and SN-38.

### *In vitro* ErbB2 silencing alters hCNT1 and hCNT3 expression in PC cells

**Figure 2:**
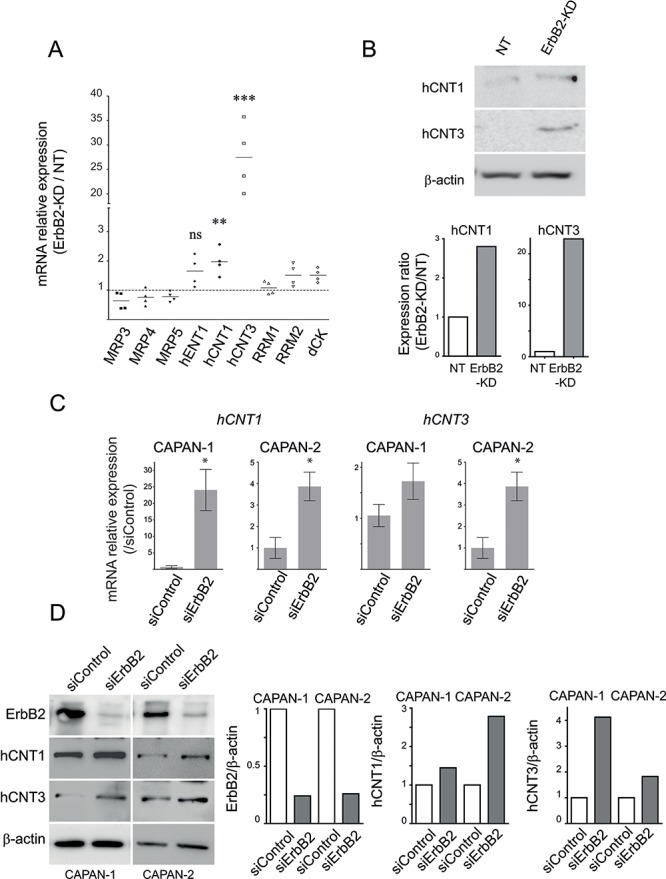
hCNT1 and hCNT3 promote gemcitabine sensitivity in ErbB2-KD cells **A.** mRNA expression of *MRP3*, *MRP4*, *MRP5*, *hENT1*, *hCNT1*, *hCNT3*, *RRM1*, *RRM2* and *dCK* was analyzed in NT and ErbB2-KD cells by qRT–PCR. The histogram represents the ratio of their expression in ErbB2-KD compared with NT cells. **B.** hCNT1, hCNT3 and β-actin expression was analysed by western blotting. Bands were quantified by densitometry and shown in the histogram. Three independent experiments were performed. **C–D.** CAPAN-1 or CAPAN-2 cells were transfected with transient ErbB2 or control siRNA. *hCNT1* and *hCNT3* mRNA or protein levels was evaluated by qRT-PCR (C) or western blotting (D), respectively. The histogram represents the ratio of ErbB2, hCNT1, hCNT3 or β-actin expression in siErbB2 compared with siControl cells.

Expression of Equilibrative/Concentrative Nucleoside Transporters (hENT1, hCNT1/3), deoxycytidine kinase (dCK), ribonucleotide reductase (RRM1/2) and Multidrug-Resistance Proteins (MRP3/4/5) was evaluated by qRT–PCR. As shown in Figure 2A, CAPAN-2 ErbB2-KD cells strongly overexpressed *hCNT3* mRNA (25-fold) and at a lower extent *hCNT1* (2-fold). Increased expression of hCNT1 and hCNT3 was confirmed at the protein level by western blotting (Figure 2B). Moreover, transient inhibition of ErbB2 led to an increase of hCNT3 mRNA and protein levels in both CAPAN-1 and CAPAN-2 cells (Figure 2C and 2D) and to an increase of hCNT1 mRNA and protein only in CAPAN-2 cells (Figure 2D). CAPAN-1 cells expressed a high basal protein level of hCNT1. Altogether, this suggests that ErbB2 inhibition promotes gemcitabine sensitivity *via* hCNT1/3 transporters.

### ErbB2 and SN-38 metabolism

**Figure 3:**
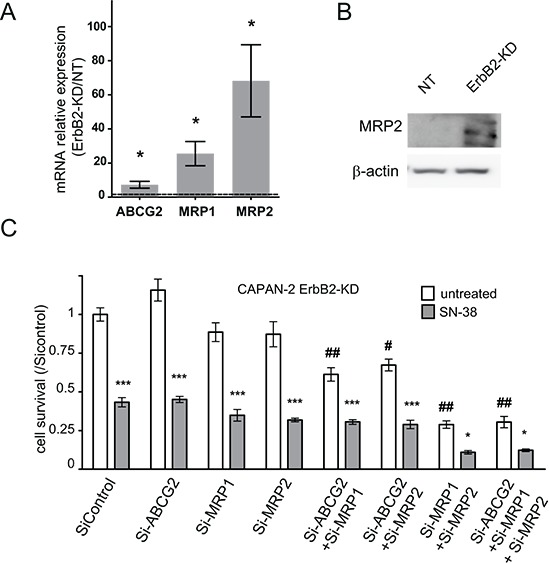
Expression of multidrug resistance related proteins (MRP)-1/2 and ABCG2 channels in CAPAN-2 ErbB2-KD cells **A.** mRNA expression of *MRP1*, *MRP2*, *ABCG2* and *GAPDH* was analyzed in NT and ErbB2-KD cells by qRT–PCR. **B.** Western blots were performed to analyze the expression MRP2 and β-actin in CAPAN-2 NT and ErbB2-KD cells. The density of each marker was measured and protein/β-actin ratio was determined and represented as histograms. Expression in NT cells was arbitrarily set to 1. Three independent experiments were performed. **C.** CAPAN-2 cells were transfected with transient MRP1, MRP2, ABCG2 or control siRNA, and then treated with SN-38. Survival rate was measured after 24 h of SN-38 treatment (20 nM) using the MTT assay. Four independent experiments were performed. * (*p* < 0.05) and *** (*p* < 0.001) indicate statistical significance compared to the corresponding untreated condition. # (*p* < 0.01) and ## (*p* < 0.001) indicate statistical significance compared to untreated control siRNA condition.

In order to evaluate the impact of the loss of ErbB2 on SN-38 metabolism, expression levels of *MRP1*, *MRP2*, *MDR1* or *ABCG2* mRNA were measured by qRT-PCR in CAPAN-2 ErbB2-KD cells compared to NT control cells (Figure 3A). CAPAN-2 ErbB2-KD cells strongly overexpressed mRNA of *ABCG2* (7-fold, *p = 0.0207), *MRP1* (25-fold, *p = 0.0126)) and *MRP2* (68-fold, *p = 0.0192). *MDR1* mRNA could not be detected. Strong overexpression of MRP2 protein was confirmed at the protein level (Figure 3B). Level of *MRP2* mRNA was increased in both CAPAN-1 and CAPAN-2 cells following transient inhibition of ErbB2 by siRNA suggesting a transcriptional regulation of MRP2 expression (Figure 5C). We also observed that transient ErbB2 silencing led to *MRP1* mRNA increase in CAPAN-1 cells whereas *ABCG2* mRNA was increased in both CAPAN-1 and CAPAN-2 cells (supplemental Figure S3). Finally, implication of MRP1, MRP2 and ABCG2 in SN-38 cell sensitivity was investigated using specific siRNAs (Figure 3C). Knocking down of MRP1 or MRP2 combined with ABCG2 reduced the survival rate of ErbB2-KD CAPAN-2 cells that harbour an increased expression of these channels (Figure 3C) as well as in CAPAN-1 and CAPAN-2 cells (not shown). Interestingly, MRP1 and MRP2 combined inhibition led to a synergistic reduction of cell viability (##*p* < 0.01) whereas subsequent inhibition of ABCG2 along with MRP1 and MRP2 did not have any additional effect. SN-38 treatment led to a 50% reduction of cell viability in ErbB2-KD cells transfected with control siRNA. SiRNA targeting MRP1 and MRP2 potentiate the SN-38 cytotoxic effect (less than 10% of alive cells, *p > 0.05).

### ErbB2 and chemosensitivity to gemcitabine and SN-38 *in vivo*

**Figure 4:**
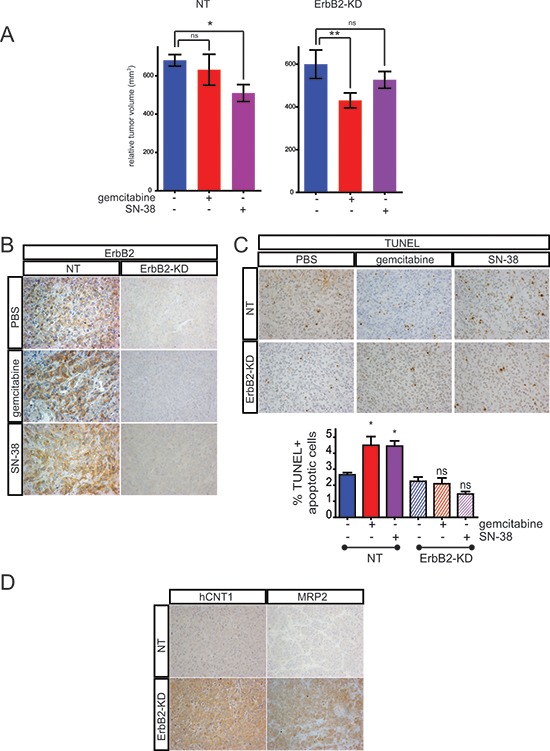
Loss of ErbB2 mediates chemosensitivity to gemcitabine and SN-38 resistance *in vivo* **A.** NT or ErbB2-KD Capan-2 cells were xenografted subcutaneously into SCID mice and the developed tumors (250 mm^3^) were then treated with either biweekly intra-peritoneal injection of gemcitabine, SN-38 or PBS for 25 days. Tumor growth was evaluated during the chemotherapy and tumor volume was calculated (*p* < 0.05 *, *p* < 0.01 **). Results are expressed as means of tumor volume. **B.** IHC analysis of ErbB2, on extracted xenografted tumors. **C.** TUNEL assays were performed on ErbB2-KD and NT xenografted tumors treated with gemcitabine, SN-38 or PBS. Results are expressed as percentage of TUNEL^+^ apoptotic cells. **D.** IHC analysis of hCNT1 and MRP2 on extracted xenografted tumors.

In order to confirm *in vivo* the effects of ErbB2 silencing on PC cell chemosensitivity to gemcitabine and SN-38, SC xenografts with CAPAN-2 NT or ErbB2-KD cells were carried out in scid mice. SN-38 treatment led to a significant decrease (*p* < 0.01) of the NT tumor volume after 25 days of chemotherapy (Figure 4A and supplemental Figure S4). Gemcitabine did not alter the tumor volume of NT xenografts. On the contrary, gemcitabine reduced the tumor volume of ErbB-KD xenografts compared to PBS control treatment (*p* < 0.05). Additionally, SN-38 treatment did not reduce the ErbB2-KD xenografts tumor volume as previously observed on NT xenografts. By IHC, we confirmed the loss of ErbB2 in ErbB2-KD xenografted tumors (Figure 4B). ErbB2 expression in both NT and ErbB2-KD tumors was not altered by gemcitabine or SN-38 treatment. We further evaluated apoptosis in xenografts by performing TUNEL assays. We observed a significant increase of TUNEL positive apoptotic cells in CAPAN-2-NT xenografts treated with either gemcitabine or SN-38 (*p < 0.05) (Figure 4C). On the contrary, percentage of TUNEL^+^ cells was not altered in ErbB2-KD xenografted tumors. Altogether, these results suggest that the loss of ErbB2 impairs gemcitabine resistance and SN-38 sensitivity of CAPAN-2 PC cells *in vivo*. Finally, we observed an increased expression of hCNT1 and MRP2 in CAPAN-2 ErbB2-KD xenografts (Figure 4D). HCNT1 and MRP2 expression were not altered by gemcitabine or SN-38 treatment (not shown).

Altogether, these results suggest that gemcitabine sensitivity could be related to hCNT1 overexpression in ErbB2-KD CAPAN-2 xenografts whereas SN-38 resistance of ErbB2-KD tumors could be related to resistance to cell death and MRP2 overexpression.

### Identification of the signaling pathways mediating SN-38 resistance

**Figure 5:**
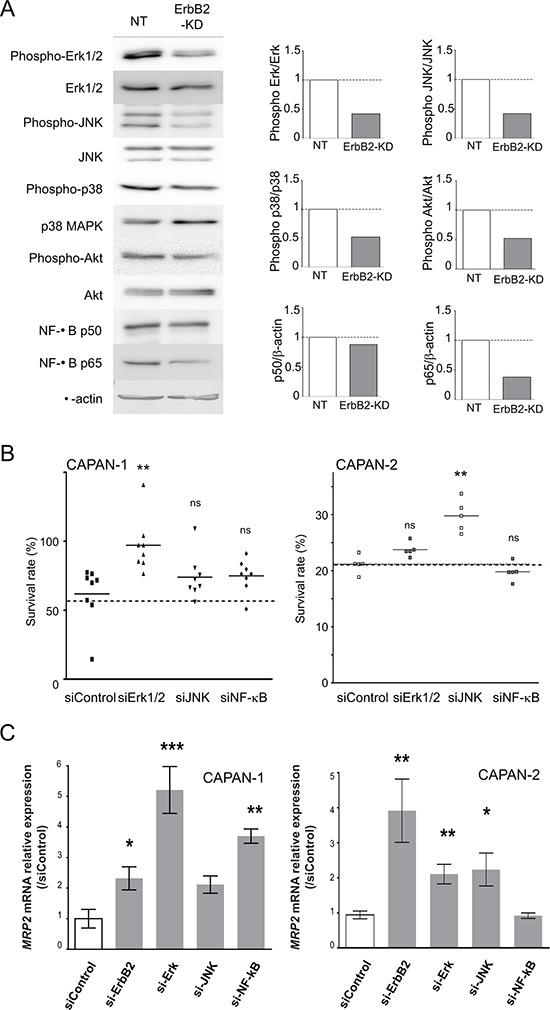
Identification of the signaling pathways involved in resistance to SN-38 in ErbB2-KD cells and MRP2 expression regulation **A.** Western blots were performed to analyze the expression and phosphorylation of ERK1/2, JNK, p38, NF-κB, Akt and β-actin in CAPAN-2 NT and ErbB2-KD cells. The density of each marker was measured and phosphorylated/constitutive or protein/β-actin ratio was determined and represented as histograms. Expression in NT cells was arbitrarily set to 1. Three independent experiments were performed. **B.** Cell survival rate was measured following Erk1/2, JNK and NF-κB inhibition in CAPAN-1 and CAPAN-2 cells using specific siRNA during 48 h before SN-38 treatment. **C.** CAPAN-1 and CAPAN-2 cells were transiently transfected with ErbB2, Erk1/2, JNK, NF-κB or control siRNA for 48 h. qPCR were performed to analyze the expression of MRP2.

We focused our studies on MRP2 transporter, for which we confirmed its overexpression in ErbB2-KD xenografts. In order to identify the molecular mechanisms that control MRP2 expression, we investigated alterations of the main signaling pathways in CAPAN-2 ErbB2-KD cells compared to NT control cells. The results indicate that the loss of ErbB2 led to a decrease of both Erk1/2, JNK, p38 MAPK and Akt activation, and a decrease of NF-κB p65 expression (Figure 5A).

Implication of Erk1/2, JNK and p38 kinases, and NF-κB and Akt pathways in SN-38 resistance was then investigated using specific siRNAs in CAPAN-2 and CAPAN-1 cells. Our results indicate that JNK targeting led to an increase of CAPAN-2 survival rate when treated with SN-38 compared to control siRNA (**p < 0.001) (Figure 5B). Erk1 targeting led to an increase of CAPAN-1 survival rate under the same conditions (**p < 0.001) suggesting cell specificity of signaling pathways regulating cell cytotoxicity (Figure 5B). SiRNA targeting NF-κB, Akt or p38 did not alter SN-38 resistance (not shown). Altogether, our results indicate that Erk and JNK regulate SN-38 cytotoxicity in CAPAN-1 and CAPAN-2 cells.

Our results indicate that transient inhibition of *ErbB2*, *Erk1/2* and *JNK* using siRNA led to a significant increase of *MRP2* mRNA expression in CAPAN-2 cells (4-, 2.1- and 2.3-fold, *p* < 0.01, *p* < 0.01 and *p* < 0.05, respectively). The same effect was observed in CAPAN-1 except for JNK (Figure 5C). Altogether, our results indicate that Erk, JNK and NF-κB negatively regulate MRP2 expression in PC cells.

### ErbB2, hCNT1, hCNT3, ABCG2, MRP1 and MRP2 in PDAC datasets

**Figure 6:**
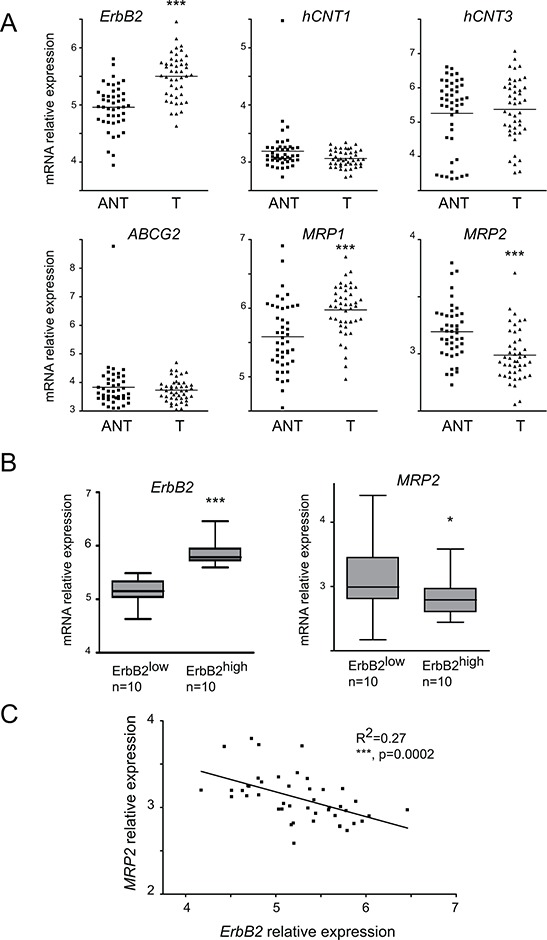
*ErbB2, hCNT1, hCNT3, ABCG2, MRP1* and *MRP2* mRNA level in human tumor tissues (T) and adjacent non tumor (ANT) tissues **A.** *ErbB2*, *hCNT1*, *hCNT3*, *ABCG2*, *MRP1* and *MRP2* mRNA expression was evaluated in PDAC datasets of the NCBI Gene Expression Omnibus (GEO, GSE28735) database to analyze whether the mRNA level differed between normal and tumor tissues. Statistical analyses were performed using paired *t*-test. **B.** *ErbB2* and *MRP2* mRNA level in 10 ErbB2^high^ and 10 ErbB2^low^ patients **C.** correlation of *MRP2* and *ErbB2* mRNA levels in 45 tumor samples. Statistical analyses were performed using Pearson's correlation coefficient (*R*^2^ = 0.27, *p* = 0.002).

Using PDAC datasets of the NCBI Gene Expression Omnibus (GEO, GSE28735) database, *ErbB2*, *hCNT1*, *hCNT3*, *ABCG2*, *MRP1* and *MRP2* mRNA expression was measured in 45 tumors (T) and adjacent non tumor tissues (ANT). We observed that *ErbB2*, *MRP1* and *MRP2* expression significantly differed (***p < 0.001) with *ErbB2* and *MRP1* increased in tumor tissues whereas *MRP2* mRNA level was reduced (Figure 6A). We confirmed these alterations in GSE16515 dataset (not shown). The tumor samples exhibiting the highest (ErbB2^high^, *n* = 10) or the lowest (ErbB2^low^, *n* = 10) mRNA level for *ErbB2* were sorted (***p < 0.001). We found that *MRP2* mRNA level was significantly reduced in ErbB2^high^ tumors (*p = 0.0239) compared to ErbB2^low^ tumors (Figure 6B). Moreover, we observed a mild converse correlation between *ErbB2* and *MRP2* mRNA overall expression in the 45 tumors collection (*R*^2^ = 0.27, *p* = 0.0002) (Figure 6C).

## DISCUSSION

Resistance to chemotherapeutic drug treatment is a major challenge for clinicians in pancreatic cancer because it is responsible for the inefficiency of therapies. The characterization of chemoresistance molecular mechanisms and identification of new therapeutic tools and/or prognosis biomarkers for personalized chemotherapy shall promote more successful therapy.

Our results show for the first time that the loss of ErbB2 sensitizes pancreatic cancer cells to gemcitabine treatment. We also show that hCNT1 and hCNT3 expression are upregulated. Previous work showed that ErbB2 inhibition improved the cytoxic effect of gemcitabine in prostate cancer cells [17]. We had also previously showed that the human mucin MUC4 forms a complex at the membrane with the oncogenic receptor ErbB2 [12] and that loss of MUC4 in pancreatic cancer cells led to an increased sensitivity to gemcitabine and an increased expression of hCNT1 correlated to cell survival [13]. Altogether, this supports the hypothesis of ErbB2 and MUC4 acting as a complex to maintain a drug resistant phenotype.

The loss of ErbB2 in CAPAN-2 pancreatic cancer cells induces an increase of SN-38 chemoresistance. In gastric cells, combination of trastuzumab (monoclonal antibody targeting ErbB2) and SN-38 effects differ depending on the order of trastuzumab and SN-38 administration. Sequential addition of SN-38 and trastuzumab increases the effectiveness of treatment while the reverse (trastuzumab and SN-38) reduces the efficiency of SN-38 [18]. In the present work, treatment with SN-38 follows the ErbB2 inhibition mimicking the sequence trastuzumab/SN-38 leading to increased resistance.

SN-38 is a topoisomerase I inhibitor displaying antiproliferative effect by stabilizing topoI-DNA complexes [19]. The action of SN-38 is optimal during the replicative phase of the cell cycle (S-phase). The loss of ErbB2 triggers a decrease of cell proliferation characterized by a decrease of the S phase and G2/M and an increase in the G1 phase [12] and could therefore reduce the efficiency window of SN-38 cytotoxicity. Recently, p53 mutant has been associated with gemcitabine chemoresistance in PDAC [20] highlighting the relevance of the p53 status on the PDAC cell response. Indeed, we observed a decreased phosphorylation of p53 in ErbB2-KD cells that are more sensitive to gemcitabine. Moreover, p53 status is closely connected to an autophagy role in pancreatic tumor development [21–23]. ErbB2 expression suppresses autophagy in mammary tumorigenesis [24] suggesting a similar complex interplay involving ErbB2, p53 and autophagy in pancreatic cancer.

In this report, we show that NF-κB, Erk and JNK pathways are altered following the loss of ErbB2 in PC cells and are involved in the complex regulation of MRP2 channel. We previously showed a link between these pathways using pharmacological inhibition of the MAPK and JNK pathways that led to a strong decrease of NF-κB expression [13]. JNK and ERK are implicated in the SN-38 resistance in a cell specific manner. Notably, CAPAN-2 cells, which express ErbB2, harbor an active JNK signaling pathway. JNK signaling pathway role in cell survival remains controversial as JNK orchestrates or antagonizes cell proliferation/survival balance in a stimuli- and tissue-specific manner [25]. JNK activation was shown to be needed in the cytotoxic effect of SN-38 in myeloma [26] or glioblastoma [27].

We also observed that Erk promotes PC cell survival in response to SN-38 treatment. On the contrary, in colon cancer cells, treatment with an Erk inhibitor enhanced SN-38 antitumor efficacy [28] indicating the cell-specific role of Erk signaling pathway in chemotherapy response. Indeed, in our work, we observe the involvement of either JNK or Erk signaling pathways that is PC cell-dependent.

We also previously showed that NF-κB regulates the hCNT1 transporter and mediates gemcitabine resistance [13]. NF-κB also transcriptionally represses the expression of MRP2 but is not involved in the cytotoxicity to SN-38 treatment in PC cells.

Altogether, these data suggest that JNK, Erk and NF-κB may not be attractive targets for overcoming drug resistance because of the complex interplay between them and their cell-specificity in cytotoxicity. However, c-fos (activated by Erk) and c-Jun (activated by JNK) form the downstream AP-1 complex activating the target genes. We propose that targeting either the upstream ErbB2 receptor or the downstream AP-1 transcription factor, should overcome these limitations.

The human genome contains 48 genes that encode ABC transporters. The overexpression of these drug efflux transporters confers a high degree of resistance to various anticancer drugs [29]. We observed that mRNA levels of *MRP1* and *MRP2* are altered in tumors compared to adjacent non tumoral tissue.

CAPAN-2 ErbB2-KD cells strongly overexpressed mRNA of *ABCG2*, *MRP1* and *MRP2* transporters. ABCG2 has been described as directly involved in acquired resistance to SN-38 in colon cancer cells [30, 31]. Mohelnikova-Duchonova *et al* described the upregulation of *ABCG2* mRNA in PDAC and proposed that ABCG2 in tumors may contribute to the generally poor treatment response [32]. However, we never observed the protein expression of ABCG2 in PC cells. MRP1 and MRP2 are two other important multidrug resistance transporters as their substrates include many commonly used chemotherapeutic drugs including SN-38 [8]. MRP2 correlates with unfavorable prognosis in a variety of tumors such as gallbladder carcinoma or breast cancer [33, 34]. MRP1 and MRP2 were previously shown to be expressed in 83 and 91% of a PDAC cohort [35]. We propose that MRP1 and MRP2 contribute to PDAC resistance to SN-38 treatment. The aberrant expression of these two channels in PDAC might be considered as a bad prognosis marker since they promote the irinotecan therapeutic failure.

In this work, we used subcutaneous xenografts which are a convenient model to study molecular mechanisms of resistance to chemotherapy *in vivo*. However, this model does not recapitulate the whole histopathological features of PDAC and notably the tumour microenvironment involvement. Further studies using pancreatic allografts in immune-deficient mice, orthotopic xenografts and syngenic models will be useful to address this question.

**Figure 7:**
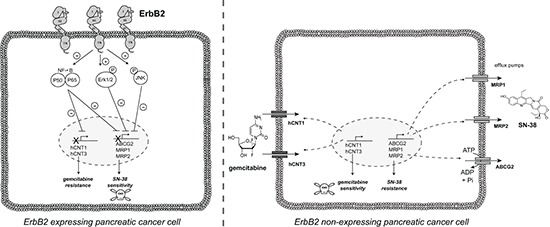
Proposed ErbB2-dependent mechanisms of PDAC cell chemosensitivity to gemcitabine and SN-38

The National Institutes of Health's (NIH) Working Group and the Biomarkers Consortium defined a biomarker as “a characteristic that is objectively measured as an indicator of normal biological processes, pathogenic processes, or a pharmacological response to a therapeutic intervention (http://www.biomarkersconsortium.org)” [36]. However, despite the discovery of thousands of cancer markers over the past decades, no new major cancer biomarkers have been approved for clinical use for at least 25 years [37] supporting that it would be more clinically relevant to optimize the use of existing markers such as ErbB2, rather than describing new ones. Our *in vitro* results show that ErbB2 expression in PC cells is associated with gemcitabine resistance and SN-38 increased sensitivity (Figure 7). We propose that ErbB2 could be a potential marker of chemotherapy response to stratify patients eligible for either gemcitabine or FOLFIRINOX treatment. Additional preclinical studies are requested to validate this hypothesis.

## MATERIALS AND METHODS

### Cell culture

CAPAN-1, CAPAN-2, BxPC-3 and Panc1 PC cell lines were cultured as previously described [38, 39]. ErbB2-knocked down (KD) cells were obtained following stable transfection of CAPAN-2 cells with pGeneClipTM puromycin vector encoding ErbB2 ShRNA (SA BiosciencesTM) as previously described [12]. The empty vector was used to raise control clones called Non Targeting (NT). Four selected clones of NT and ErbB2-KD cells were pooled in order to avoid clonal variation. All cell lines were periodically authenticated by morphologic inspection, expression profile of ErbB2 by western blotting and tested negative every 6 months for mycoplasma contamination using Mycoalert kit (Lonza). All cells were maintained in a 37°C incubator with 5% CO_2_ and cultured as the parental cells.

### Western-blotting

Total cellular extracts and western blotting were performed as previously described [40, 41] using antibodies against phospho-Erk1/2 (Thr202/Tyr204) (clone 20G11, 1/500), Erk1/2 (clone I37F5, 1/500), NF-κB Phospho-p65 (Ser536) (clone 93H1, 1/500), NF-κB p65 (clone E498, 1/500), phospho-SAPK/JNK (Thr183/Tyr185) (9251, 1/500), SAPK/JNK (clone 56G8, 1/500), p53 (9282, 1/500), phospho-p53 (Ser15) (9284, 1/500), from Cell Signaling Technology (Ozyme, Saint Quentin Yvelines, France), hCNT1 (clone H-70, 1/200), NF-κB p50 (H-119, 1/500), MRP2 (H-17, sc-5770, 1/500), Bax (N-20, 1/500), BclXL (H-5, 1/500) from Santa Cruz Biotechnology Inc. (Heidelberg, Germany) or hCNT3 (HPA023311; 1/500), β-actin (A5441, 1/5000) from Sigma-Aldrich (St. Quentin Fallavier, France). Antibodies were diluted in 5% (w/v) non-fat dry milk in Tris-Buffered Saline Tween-20 (TBS-T). Peroxydase-conjugated secondary antibodies (Sigma-Aldrich) were used and immunoreactive bands were visualised using the West Pico chemoluminescent substrate (Thermo Scientific, Pierce, Brebières, France). Chemo-luminescence was visualised using LAS4000 apparatus (Fujifilm). Densities of bands were integrated using Image Quant TL 8.1 (GE Healthcare Life Sciences, Velizy-Villacoublay, France) and represented as histograms. Three independent experiments were performed.

### Cytotoxicity assay

Cells were seeded in growth medium into 96-well plates at a density of 10^4^ cells per well. After 24 h incubation, the medium was replaced by fresh medium containing gemcitabine, 5-FU, oxaliplatin or SN-38 at the determined concentration (range: 10 nM–20 μM) and incubated for 72 h at 37°C. The viability of cells was determined using the 3-(4, 5-dimethylthiazol-2-yl)-2, 5-diphenyltetrazolium bromide assay (MTT, Sigma-Aldrich) as previously described [13]. For drug combinations, concentrations corresponding to 40% cell survival were used as follows: 5-FU at 1 μM, oxaliplatin at 1 μM and SN-38 at 20 nM.

### Quantitative reverse transcription polymerase chain reaction qRT-PCR

Total RNA from PC cells was prepared using the NucleoSpin^®^ RNA II kit (Macherey Nagel, Hoerdt, Germany). cDNA was prepared as previously described [42]. PCR was performed using SsoFastTM Evagreen Supermix kit following the manufacturer's protocol using the CFX96 real time PCR system (Bio-Rad). Primer information is given in Table 1. Each marker was assayed in triplicate in three independent experiments. Expression level of genes of interest was normalized to the mRNA level of *GAPDH* housekeeping gene.

**Table 1:** Primer sequences used for quantitative real-time RT-PCR

Gene	Forward Primer 5′ to 3′	Reverse Primer 5′ to 3′	Amplicon size (bp)
*dCK*	GAGAAACCTGAACGATGGTCTT	TCTCTGCATCTTTGAGCTTGC	102
*hENT1*	CTCTCAGCCCACCAATGAAAG	CTCAACAGTCACGGCTGGAA	123
*hCNT1*	CCTCACCTGTGTGGTCCTCA	AGACCCCTCTTAAACCAGAGC	86
*hCNT3*	CTTTTCTGGAGTACACAGATGCT	CGGCAGGACCTTAAATGCAAA	108
*RRM1*	CTGCAACCTTGACTACTAAGCA	CTTCCATCACATCACTGAACACT	108
*RRM2*	CCACGGAGCCGAAAACTAAAG	CTCTGCCTTCTTATACATCTGCC	131
*MRP3*	GGAGGACATTTGGTGGGCTTT	CCCTCTGAGCACTGGAAGTC	90
*MRP4*	AAGTGAACAACCTCCAGTTCCAG	GGCTCTCCAGAGCACCATCT	119
*MRP5*	AGAACTCGACCGTTGGAATGC	TCATCCAGGATTCTGAGCTGAG	104
*ABCG2*	ACGAACGGATTAACAGGGTCA	CTCCAGACACACCACGGAT	93
*MRP1*	GTCACGTGGAATACCAGCAAC	ACATGACCGAGGCTACATTCA	142
*MRP2*	ACAGAGGCTGGTGGCAACC	GTGGATCTAGAGACAGACAAC	129
*MDR1*	TTGCTGCTTACATTCAGGTTTCA	AGCCTATCTCCTGTCGCATTA	105
*GAPDH*	CCACATCGCTCAGACACCAT	CCAGGCGCCCAATACG	70

### RNA interference

Transient inhibitions of *ABCG2* (sc-41151), *MRP1* (sc-35962) *MRP2* (sc-35963), *Akt* (sc-43609) and *p38* (sc-29433) were performed using a pool of siRNA designed by Santa Cruz Biotechnology following cell transfection with Effectene^®^ (Qiagen) as described by the manufacturer. Transient KD for *ErbB2*, *Erk1* (MAPK3), *JNK1* (MAPK8), *NF*-*κB* (p105 NF-κB) was performed using siRNA from Dharmacon (Thermo Scientific) following the protocol described previously [41]. Controls were performed using a Non-Targeting siRNA (NT) in both protocols. Cells were seeded at a density of 5 × 10^5^ cells per well into 6-well plates for RNA and protein extraction, or at a density of 10^4^ cells per well into 96-well plates for cytotoxic assay, and left for 48 h at 37°C before gemcitabine treatment (100 nM) or SN-38 (20 nM) for another 24 h at 37°C.

### Confocal microscopy

Immunofluorescence was performed on ErbB2-KD and NT CAPAN-2 cells grown on Lab-Tek Chamber Slides (Nunc). The cells were fixed with 4% (v/v) paraformaldehyde for 20 min at 4 °C, quenched for 20 min with 50 mM NH_4_Cl in D-PBS+Mg^2+^+Ca^2+^ (Life Technologies) and permeabilized with 0.2% (w/v) saponin in D-PBS+Mg^2+^+Ca^2+^ for 20 min. The saturation step was performed for 20 min with D-PBS+Mg^2+^+Ca^2+^ containing 3% (w/v) BSA and 0.2% saponin. Cells were incubated overnight with an anti-phospho-Ser139 H2A.X antibody (Merck-Millipore #05-636) diluted to 1:500 in D-PBS+Mg^2+^+Ca^2+^ containing 3% (w/v) BSA and 0.2% (w/v) saponin. Alexa-633 conjugated goat anti-mouse antibody was used as a secondary antibody. F-actin staining was obtained by incubating cells with Alexa-488 conjugated Phalloidin (Molecular Probes, Life Technologies) for 20 min at room temperature. Slides were mounted in mounting medium containing diamidino-2-phenylindole and visualized with a Zeiss LSM 710 confocal microscope (Carl Zeiss Microscopy). Images were captured and analysed with the Zeiss Efficient Navigation software (ZEN, Carl Zeiss Microscopy). Three independent experiments were performed.

### Subcutaneous xenografts

NT or ErbB2-KD CAPAN-2 cells (2 × 10^6^ cells in 100 μl Matrigel) were injected subcutaneously into the flank of seven-week-old male Severe Combined Immunodeficient (SCID) mice (CB17, Charles Rivers, France). Once palpable tumors were developed (250 mm^3^), gemcitabine (15 mg/kg), SN-38 (40 mg/kg) or PBS (200 μl) were injected intra-peritoneously, twice a week. Six mice were used per group. Tumor size was evaluated by measuring the length (l) and the width (L) twice a week and tumor volume was calculated with the formula (l^2^ × L). All procedures were in accordance with the guideline of animal care committee (Comité Ethique Expérimentation Animale Nord Pas-de-Calais).

### Immunohistochemistry (IHC)

Pancreatic tissues were fixed in 10% (w/v) buffered formaldehyde, embedded in paraffin, cut at 4 μm thickness and applied on SuperFrost^®^ slides (Menzel-Glaser, Braunschweig, Germany). Manual IHC was carried out as previously described [43]. The antibodies were used as followed: anti-ErbB2 (1:200, DAKO), anti-hCNT1 (1:100, H-70), and anti-MRP2 (1:100, sc5770, santa cruz). TUNEL assay was performed using ApopTag^®^ Plus Peroxidase *In Situ* Apoptosis Kit (Chemicon) following manufacturer's protocol.

### Gene Expression Omnibus microarray

Public PC microarrays were analysed from the NCBI Gene Expression Omnibus (GEO) database (http://www.ncbi.nml.nih.gov/geo/). Two sets of gene-expression profiles from GEO containing both normal pancreas and PC tissue were used: 45 tumors and adjacent non-tumor tissues from PDAC cases (GSE28735) and 52 samples (16 had both tumor and normal expression data, and 20 only had tumor data (GSE16515). Data were analysed using GEO2R software. The dataset GSE28735 used Affymetrix GeneChip Human Gene 1.0 ST array. The dataset GSE16515 used the Affymetrix Human Genome U133 Plus 2.0 Array.

### Statistical analyses

Statistical analyses were performed using the Graphpad Prism 4.0 software (Graphpad softwares Inc., La Jolla, USA). Differences in data of two samples were analysed by the student's *t* test or ANOVA test with selected comparison using tukey post-hoc test and were considered significant for *P*-values < 0.05 *, *p* < 0.01 ** or *p* < 0.001 ***. For paired tumor and adjacent non tumor samples, paired *t* test was performed.

## SUPPLEMENTARY FIGURES


