# Supplementary material for: The oncogenic receptor ErbB2 modulates gemcitabine and irinotecan/SN-38 chemoresistance of human pancreatic cancer cells via hCNT1 transporter and multidrug-resistance associated protein MRP-2
Source: Oncotarget. 2015 Mar 26;6(13):10853–67. doi: 10.18632/oncotarget.3414 (PMC4484424; doi:10.18632/oncotarget.3414)
Supplement: Supplementary file 1 [file oncotarget-06-10853-s001.pdf]

SUPPLEMENTARY FIGURES

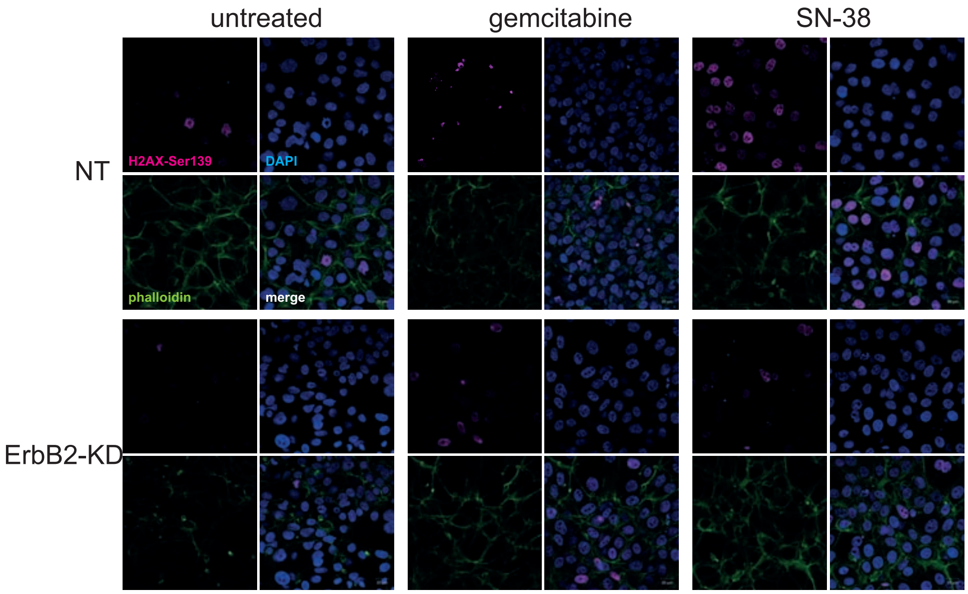

Supplementary Figure S1: phospho-Ser139 H2A.X foci in NT and ErbB2-KD CAPAN-2 cells following SN-38 and gemcitabine treatment. Phospho-Ser139 H2A.X foci were visualized by immunofluorescence and confocal microscopy in NT and ErbB2-KD CAPAN-2 cells. Cells were treated with gemcitabine (100 nM) or SN-38 (20 nM) for 24 h. F-actin was stained with Alexa-488 conjugated Phalloidin. Nuclei were stained using DAPI. Three independent experiments were performed. Scale bar = 20 µm.

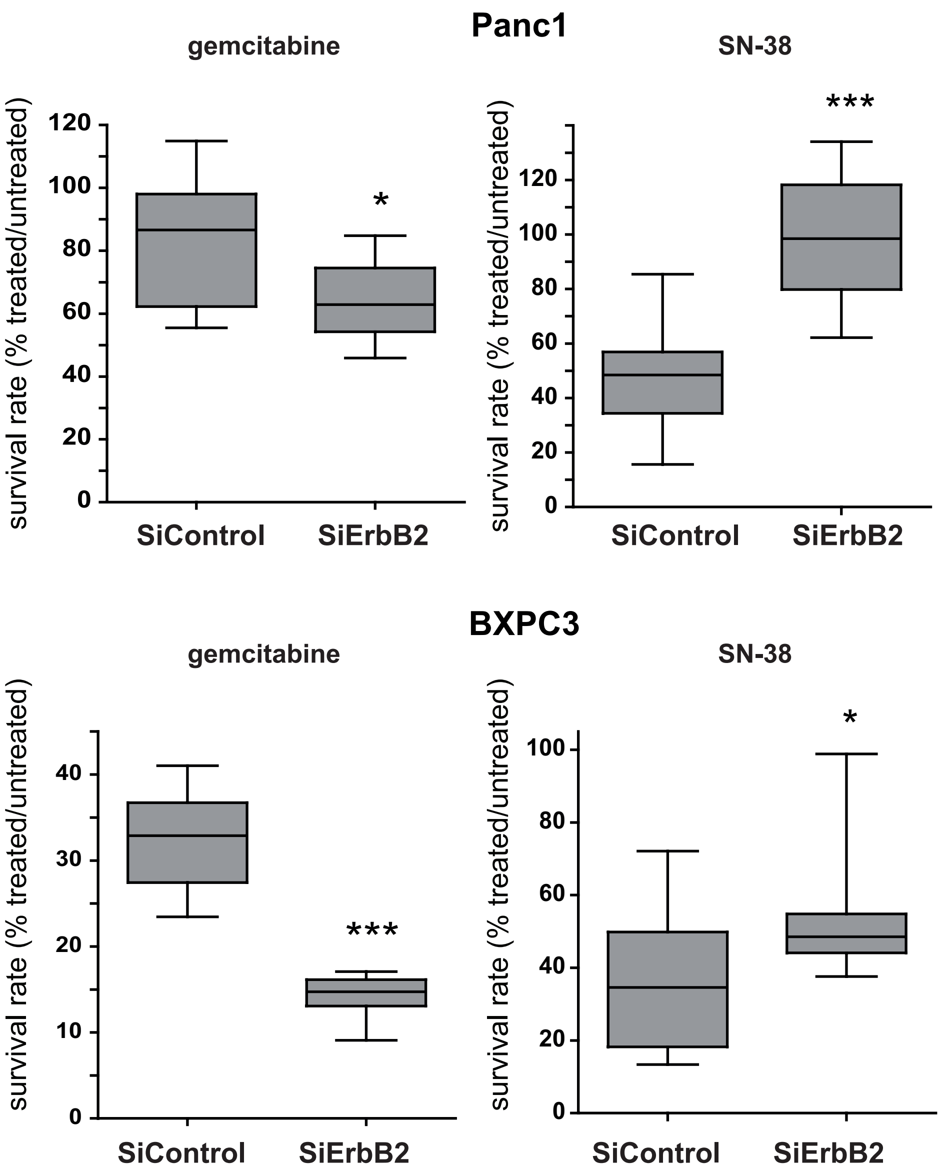

Supplementary Figure S2: ErbB2 deficient Panc1 and BxPC-3 cells are more sensitive to gemcitabine and resistant to SN-38 treatment. Survival rates in Panc1 and BxPC-3 cells were measured following transient siRNA-ErbB2 or siControl and treatment with gemcitabine or SN-38 using the MTT assay. *($p < 0.05$) and ***($p < 0.001$) indicate statistical significance compared to the corresponding untreated condition.

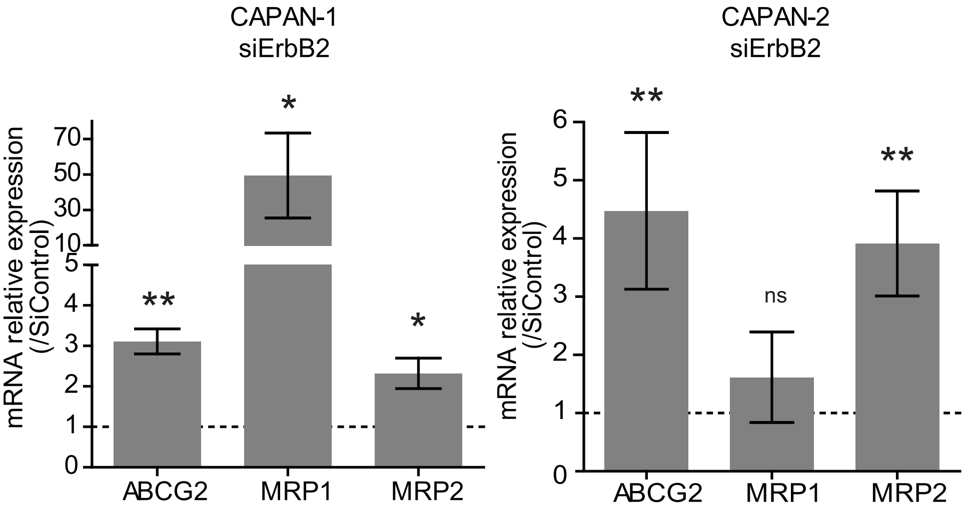

Supplementary Figure S3: Transient ErbB2 siRNA and expression of MRP-1/2 and ABCG2. CAPAN-1 and CAPAN-2 cells were transfected with transient ErbB2 or control siRNA for 48 h before RNA extraction. mRNA expression of *MRP1*, *MRP2*, *ABCG2* and *GAPDH* was analyzed in by qRT–PCR. The histogram represents the ratio of *MRP1*, *MRP2* and *ABCG2* expression in siErbB2 compared with siControl cells. *($p < 0.05$) and **($p < 0.01$) indicate statistical significance compared to the corresponding siControl condition.

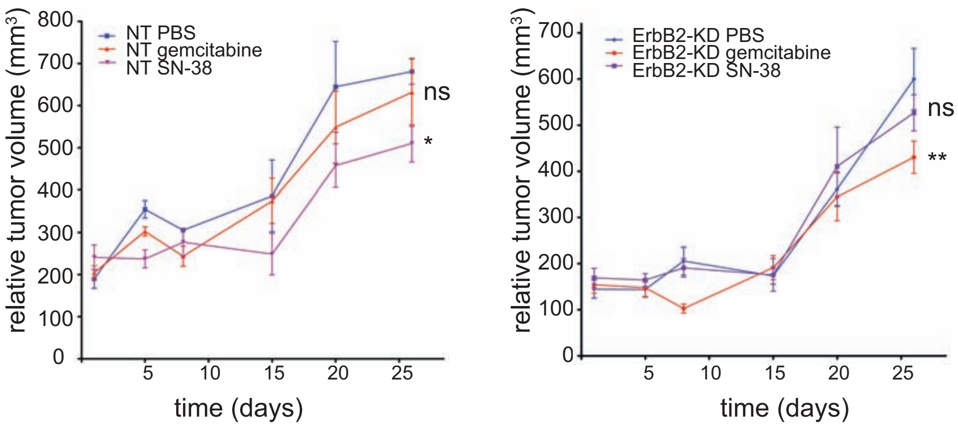

Supplementary Figure S4: Tumor volume progression of CAPAN-2 xenografts shows gemcitabine sensitivity and SN-38 resistance in ErbB2-KD tumors. A. NT or ErbB2-KD CAPAN-2 cells were xenografted subcutaneously into *SCID* mice and the developed tumors (250 mm^3) were then treated with either biweekly intra-peritoneal injection of gemcitabine, SN-38 or PBS for 25 days. Tumor growth was evaluated during the chemotherapy and tumor volume was calculated ($p < 0.05$ *, $p < 0.01$ **). Results are expressed as means of tumor volume.
